# *Välkky*’s voyage on to a hospital ward: Expectations, explorations and emergent robocentric nursing care

**DOI:** 10.1177/13634593241303610

**Published:** 2024-12-04

**Authors:** Sarah Nettleton, Nik Brown, Karl Atkin, Luna Dolezal, Sanna Metsäketo, Daniel Robins

**Affiliations:** University of York, UK; University of York, UK; University of York, UK; University of Exeter, UK; University of Eastern Finland Faculty of Health Sciences, Finland; University of York, UK

**Keywords:** ethnography, technology in healthcare, organisation of health services

## Abstract

Drawing on ethnographic fieldwork in Finland, we report on the trial of a teleoperated care robot named Välkky introduced onto a fully operational hospital neurological ward. Our data revealed a narrative arc where participants’ early expectations of the hospital-based trial altered as the project unfolded. Greeted with techno-excitement and experimental enthusiasm about the place of robotics in reshaping roles within clinical care, Välkky became the focus for collaborative in situ learning, adaptation and redesign amongst the roboticists, designers, nurses, patients, and managers. Välkky acted as an ‘attractor’ provoking thinking about, and a reimagining of, future arrangements of care. Our empirically informed insights seek to pave the way for real-world nuanced thinking that pushes beyond human/non-human and success/failure binaries. Building on debates in STS and feminist posthumanism, we propose a *robocentric approach*, which encourages us to ‘queer’ health care robots, and to understand them as fluid, hybrid, distributed and relational figures, rather than purely as inert, mechanical, non-human objects that might replace humans. Nursing care practices by and with robots will generate new meanings and practices of care that will emerge iteratively, as caring relations, relationships and practices develop within the context of operational ward environments. Robots may or may not be able support care, but they will invariably challenge what care is.

## Introduction

This paper offers a critical commentary generated during a study involving sociologists and a philosopher participating in a pilot project of a full body teleoperated care robot, named *Välkky.* Led by a commercial tech company, the trial took place in a hospital in-patient neurological ward in Finland. Originally built as a TB sanatorium, the hospital is undergoing a €1 billion upgrade and aspires to be amongst the most technologically advanced hospitals in Europe. By introducing a prototype robot onto the ward, the project involved training nurses to teleoperate the robot and co-operate with it in a hospital care setting. The exploratory pilot project sought to foster co-creative dialogue between roboticists, nurses, hospital managers, patients, philosophers and sociologists to co-design an experimental prototype and to parametrise the scope, potential, limitations, and in situ contingencies relevant to redesigning robotic applications for nursing in healthcare settings.

The question addressed in this paper is: *How did the project participants (a) respond to the introduction of Välkky into their working environment and (b) to what extent did their initial expectations alter during the study?* We analysed participants’ expectations and experiences to help us think about the practicalities of operating with care robots, and to reflect on how *Välkky’s* presence might disrupt established ideas about nursing care. Our analytic strategy, by using this data ‘to think with’ (Atkinson, 1997: 369), assesses technology ‘in its making’ (Ingold, 2013: 10). We begin by locating our analytic interpretations in the context of debates that centre on the anticipations and disenchantments with care robotics.

## Care robotics: Challenging promissory narratives

Roboticization has been lauded as a means of addressing a ‘crisis in care’ largely in the Global North (Fosch-Villaronga, 2019; Niemelä et al., 2021a; Sætra and Fosch-Villaronga, 2021). A number of studies have been designed to identify and measure discrete outcomes of care robots (Schoenhofer et al., 2019; van Wynsberghe, 2013). It is anticipated that robots will carry out quantifiably ‘menial tasks in hospital settings’ and reduce workloads (Soriano et al., 2022: 2). Robots are presumed to have the potential to ‘serve as supplemental healthcare workers in hospitals’, supporting nurses to ‘perform logistical and laborious physical tasks’ and be ‘assigned to routine tasks such as measuring patients’ vital signs’ (Christoforou et al., 2020: 2). These aspirations are consistent with techno-solutionism, an ideology that rests on the belief that social problems in healthcare can be readily identified, documented and fixed by technological innovations (Gardner and Warren, 2019; Morozov, 2013).

However, critics of techno-solutionism argue that social robotics in healthcare will invariably fail to live up to expectations (Maibaum, et al., 2022; Vallès-Peris and Domènech, 2023). For instance, Lipp (2024) undertook an ethnography of a project designed to evaluate care robotics in a ‘realistic’, simulated, domestic setting. He describes how the roboticists orchestrated ‘robot dramas’ to ‘recalibrate promissory discourses’ when desired outcomes fell short of expectations, due to the ‘precarity of the technology’. Vallès-Peris and Domènech (2023) argue that narratives which present robotics as a ‘solution’ to the ‘crises in care’ rest on a priori definitions of ‘care’ and ‘care work’ which assume that ‘care operates with the same logic as productive work’ (p. 3). However, ‘care’ and ‘care work’ are highly contested, malleable and situationally specific (Klostermann et al., 2022). Nursing scholars critical of ‘new managerialist’ agendas have long argued that the vocabulary of, ‘efficiency’, ‘outcomes’, ‘measurement’, ‘evidence-based practices’ and ‘nursing process’ gloss over the nuanced complexities of nursing care practices (Latimer, 1995; Traynor, 1996). Nevertheless, similar ‘rhetorical skills and techniques’ (Traynor, 1996: 335), which refer to task-based care are being deployed by those who call for investment into care robotics. A central trope runs through these debates. A growing demand (and need) for care, alongside a shortage of care workers, requires healthcare agencies to capitalise on robotic technologies. This enables care professionals to focus on ‘emotional and social engagement’ and complex decision-making (Christoforou et al., 2020; Darko et al., 2023; Darzi, 2018; Shilpa and Garg, 2023). This trope we argue is too crude. It is not epistemologically apposite to undertake empirical studies that seek to explicate or measure care outcomes. Instead, we should unpack how care robots operate in the ‘messiness’ (Law, 2004) and the ‘wilds’ of real life ‘social situations and cultures’ (Jung and Hinds, 2018: 2). Rather than seeking to evaluate a priori outcomes of care, our aim in this study is to identify *developing* evidence, which can pave the way for more real-world nuanced applications (Timmermans and Berg, 2003) and *temper* tropes which presume health care service settings are ‘empty spaces waiting for machines’ (Maibaum et al., 2022: 476).

Robots, as agents and practitioners of care, also invariably tap into debates on transhumanism and posthumanism and the augmentation or enhancement of humans, reflecting discussions anthropocentric in nature. A transhumanism position suggests the ‘transcending of human frailties’, with robotic technologies offers scope for ‘more physically able human beings’ who bring ‘enhanced organismic capacities to meet the demands of the future’ (Locsin et al., 2021: 8). While Locsin et al are enthusiastic about such ‘transhuman potential’ they caution against a ‘starry eyed’ technological optimism. Posthumanism by contrast, foregrounds epistemological and ontological questions such as; what does it mean to be human, and what is at stake when human embodiment becomes entangled with non-human materialities.

‘If such technologies are an irreducible facet of our posthuman world, then we should surely strive to develop a posthumanist ontology, epistemology and ethics. At very least, the notion of stable and distinctive being must give way to the transmutations of dynamic becoming in the context of multiple others, and that entails thinking through our responsibility to the relationality and interdependency of all forms of matter’ (Shildrick, 2023: 133).

This position implies a ‘queering’ of humans and robots, which by disputing distinctions between natural and artificial, organic and inorganic, human and non-human introduces the notions of assemblage to better appreciate how complex configurations of heterogeneity disrupt notions of singularity and the singular self (Shildrick, 2023). The significance to our thinking about care robotics is that the posthuman position challenges an anthropocentric perspective, which posits that ‘real’ or ‘authentic’ care can only be done by humans, and that face-to-face human care is invariably the ‘gold standard’ (DeFalco, 2020: 50).

These arguments disrupt the medical sociological canon where care is conceptualised as, ’body work’ involving ‘direct, hands-on activities’ such as, ‘assessing, diagnosing, handling, treating, manipulating, and monitoring bodies’ (Twigg et al., 2011: 171), requiring ‘a physical co-presence’ (Cohen, 2011). Post-humanist feminists challenge the idea that human-to-human care is superior to mediated contact (Barad, 2003; Braidotti, 2013; Hayles, 1999), establishing a position that frees us up to ask:

Could care robots open opportunities for post humanist posthuman care, that is, care that works with and from a non-anthropocentric vision of human/non-human relations, a vision of care based instead on difference, hybridity and perpetual becoming? (DeFalco, 2020: 49)

Drawing on these arguments we call for a non-anthropocentric vision of care. We refer to this as a *robocentric approach*, which by queering the relationship between robots and humans, encourages us not to see robots as purely inert, mechanical objects but instead as fluid, hybrid and relational figures. Care robots are invariably hybrid; they are assembled by humans and machines, they are technologically augmented to do care practices, and they are fluid hybrids that change over time and place. As we go on to demonstrate, in health care settings they can come to take on ‘human’ qualities of liveliness, sociability, and expressivity. Not looking to robots to replace or replicate humans, a *robocentric approach* seeks to think about robots as actors whose form and capacities for care are continually and inexorably taking shape. We followed *Välkky* onto the hospital ward to generate and provide empirical texture to our thinking. The original contribution of our study lies in its empirical and conceptual novelty. We present an empirically informed discussion underpinned by unique data generated during a world-first trialling of the teleoperated care robot *Välkky* in a hospital setting to show how care robots are hybrid in hospital sites and how a *robocentric approach* offers an innovative conceptual resource for future research.

## Study design and methods

**Image 1.**
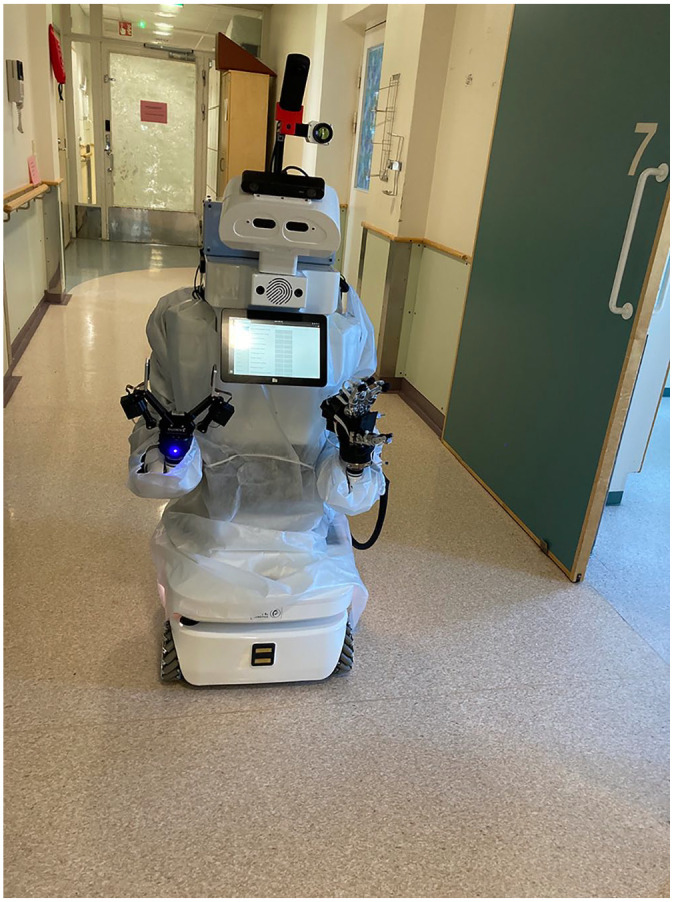
Välkky gowned.

**Image 2.**
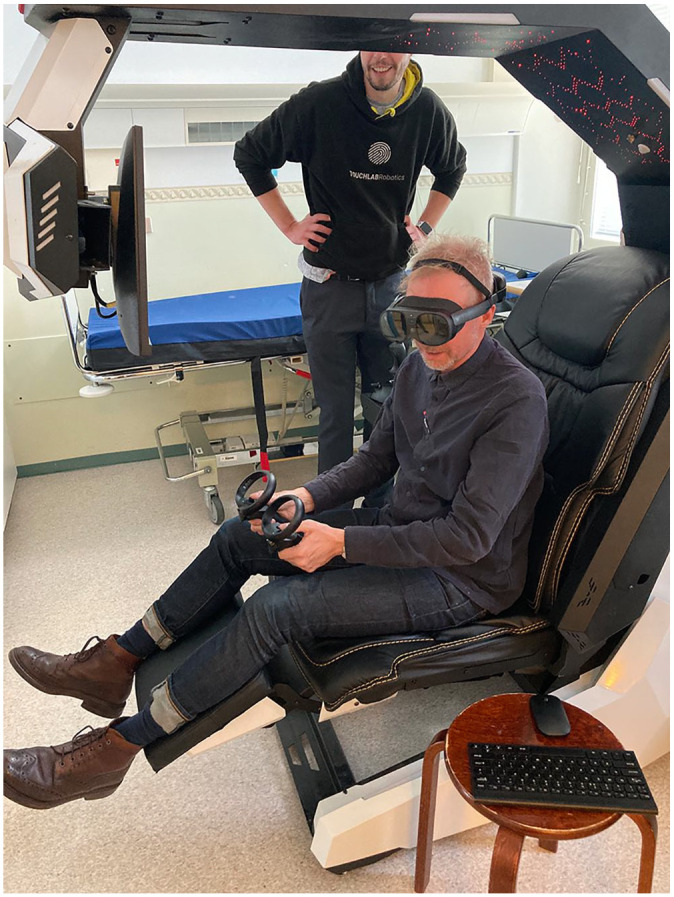
Teleoperator with trainer.

The project comprised a collaboration between a UK deep-tech startup and academics from the humanities and social science trialling this world-first teleoperated robot in a hospital ward setting (see: Välkky Healthcare Robot (2023). The management of the hospital were keen to host the trial in preparation for their relocation to a state-of-the-art, high-tech hospital being built on an adjacent site. Two wards were dedicated to the project. A general ward, empty of patients but full of the paraphernalia of a hospital ward (e.g. trollies, beds, wheelchairs, IV drips), and a fully functioning neurological ward treating patients recovering from stroke. The roboticists delivered hands-on training for nurses, to teach them how to operate *Välkky* on both the non-operational ‘practice’ ward and the fully operational one. The nurse operators were seated in a Scorpion Chair, ergonomically designed to minimise neck or back strain and also to enhance a sense of immersion in the technologically mediated clinical world. A Virtual Reality (VR) headset enabled further immersion with 360° visual input. Nurses used joystick controls to move *Välkky* remotely, backward, forwards and sideways. *Välkky* has a ‘robotic’ anthropomorphic form with a torso, arms, hand, fingers, eyes and head which moved in unison with the nurse’s head movements. See Image 1 *Välkky gowned* and Image 2 *Teleoperator with trainer.*

Data collection included fieldwork observations on the wards during training sessions, conversations with key informants (roboticists, designers, nurses, and patients); focus group discussions with nurses (*n* = 3); and semi-structured interviews with nurse operators (NO) (*n* = 6), nurse managers (*n* = 2); and one patient. Six patients agreed to participate but due to deterioration of symptoms, unanticipated hospital discharge and/or medical procedures, five were unable to be interviewed. Informal conversations with patients, however, were included in the fieldwork notes. The three focus groups were conducted on the first site visit when training was just starting (May 2023). The nine interviews were conducted some months later (August 2023). The nurses had previously been introduced to the teleoperated robot at an event in a space located beneath a large shopping mall in the city. Here a network of rooms comprised an architectural design template for the new hospital. Ethical approval did not enable us to interview doctors. This is a limitation of the study.

The researchers (one British (SN), one Finish (SM)) observed training sessions – in a mix of English and Finnish– usually lasting approximately 1 hour. The researchers also followed *Välkky* as it was teleoperated by a trainee nurse on the operational neurological ward and conducted qualitative face-to-face interviews. Participants were asked which language they would prefer for the interview. Most chose English, although two interviews were conducted primarily in Finnish. Further meetings with the engineers prior to and after the site visits provided updates on progress, hitches, and developments. Data collection was carried out in varying sites including: the hospital wards, the site of the prototype of the hospital under construction and the training laboratory. The UK deep-tech startup company and the City of Helsinki Finland secured ethical approvals (*Helsinki Helsingfors - Research permit application Ansökan om Aorskningstillstånd Social and health care, Social and health sector* 14/12/2022). Informed consent was obtained from all participants.

During the focus group discussions and interviews, participants were asked inter alia about: their expectations of the robot; experiences of operating or working alongside it; and views on the potential merits and demerits of care robotics for their work. In the second round of interviews, nurses were asked to describe how they found operating and working with *Välkky*. Recorded interviews were transcribed and along with field notes, uploaded into a secure shared drive, accessible to all members of the team. Analysis comprised familiarisation of the data where the philosopher and the sociologists independently summarised recurrent issues. The data were then coded to facilitate retrieval. Themes were identified iteratively through discussion informed by the research team’s expertise in STS, phenomenology, embodiment, and health care policy.

Our analytic strategy was one of ‘abductive analysis’ whereby data are used to ‘think with’:

‘At root, the important thing for the ethnographer or other qualitative researcher is that data should be things to think with and to think through; there is, or should be, a constant shuttling between ideas and data, data and ideas (Atkinson, 2018: 415).

The iterative process of shuttling back and forth between data, ideas and concepts goes to the heart of the qualitative methodology which is fundamentally a creative dialectic between data and theory (Tavory and Timmermans, 2014). As opposed to ‘grounded theory’ which delves deeply into the data to create theory from empirical material, abductive analysis works *with* related conceptual literatures. As Timmermans and Tavory (2012) put it, empirical findings are best reflected upon ‘against a background of multiple existing sociological theories’ (p. 169). This approach is especially instructive when thinking with robotic technologies (Ingold, 2013) to contribute to their co-design and co-construction for care. We present our findings to follow the chronology of the project and to reveal how expectations and sociotechnical imaginaries (Jasanoff and Kim, 2015) adjusted as participants got to know Välkky. A narrative arc emerged, where we saw how nurses greeted *Välkky* with a degree of techno-enthusiasm and excitement, which later gave way to some disappointment and despondency, followed by a rejuvenated commitment to, and a growing fondness of, the robot. It was through our analytic interpretation of these data that we came to formulate our notion of *robocentricism*; we came to appreciate how the study participants’ expectations shifted as they moved from describing imaginaries of ‘robots’ to engaging with *Välkky* in practice. Rhetorical statements on care robots in the initial interviews gave way to descriptions of the realities of working with a robot. A robot who they came to care about and were keen to find ways to care with.

## Welcoming Välkky

### Techno-excitement and (ambivalent) experimental enthusiasm

During the initial focus group discussions, nurses spoke with enthusiasm about the trial and the robot. They saw their involvement in this innovative ‘high-tech’ research as a privilege (cf Henderson, 1998).

‘We are proud because from a lot of places they have chosen Helsinki and have chosen [named] hospital and so we are proud to be the first to try this new technology’ (No4). ‘I think its wider than that; I feel privileged to be part of this because it could add to the future, it feels good to help’ (No1) (FG 1).

The Head of Nursing Director (HND) said: ‘We want to show that [named hospital] is progressive and to show that we have new possibilities, and we are willing to do something new’. A statement that echoes Finnish health policy which is committed to integrating robotic technologies into health and welfare services (Niemelä et al., 2021b). The HND recalled:

‘We have a senior person in our organisation who asked: “Could we get some robots or something like that in our hospital and are we interested?” We said, “yes, yes absolutely”’.

She explained how much of the work done by nurses does not require ‘advanced nursing qualifications’ because it is ‘repetitive’ and ‘unskilled’; a view which resonates with normative neoliberal discourses of care, conceived of as task-based activities (Latimer, 1995; Maibaum et al., 2022; Traynor, 1996).

Although imaginaries of technological advancement were to the fore, other visions or ‘contested futures’ (Brown et al., 2000) were also evident. During focus group discussions, when the interviewer commented that all staff seemed enthusiastic about *Välkky*, the participants laughed:

Some people had a bad reaction, they said that robots are coming to replace our jobs (No3). I think there are some who are concerned about the money, and on a nurses’ salary they see it [the robot] costing a lot; spending money for this but not nurses salary (No4) (FG 1).

Participants, by reporting views voiced by their colleagues, provided evidence of alternative opinions, opinions which echo imaginaries of dystopian futures built around narratives of labour replacement arising from AI, robotics, automation and autonomous systems. A nurse ward manger (NWM) also told us that some of her colleagues were concerned that ‘the robot will take our jobs’, something she said was ‘laughable because there is such a shortage of staff!’ (NWM01). Nevertheless, the view is consistent with the sociology of expectations that shows how narratives of wholesale substitution of workers are characteristic features of misplaced technological determinism and ‘hype’ (Borup et al., 2006).

Competing and ambivalent views on nursing work were also articulated by our participants. Delegable tasks were presumed to require little in the way of cognitive or emotional skill (Bedaf et al., 2019). This notion that care-work can be broken down into discrete tasks reflects the imaginaries of roboticists interviewed by Vallès-Peris and Domènech (2020: 613), who report their participants as saying that robots could take on the ‘boring’, ‘tedious’, ‘time consuming’ ‘repetitive’, and ‘heavy’ tasks to ‘free caregivers’ to carry out more meaningful and rewarding work. Others we spoke to, however, were more circumspect. For them the idea of care robots was seen as anathema to nursing, which as one nurse put it is ‘a human profession’ (No3: FG2). Another nurse told us that telepresence could never replace human-to-human co-presence:

‘A nurse really needs to be present with the patient and feel the patient’s feelings and emotions to offer support to patients. [INT: So even though it is a human nurse teleoperating the robot you think it is not the same?] I think you could be going through the ward talking to patients, maybe giving them a glass of water but still it doesn’t feel, it’s not the same as a human nurse. One reason is that when you operate the robot you have to concentrate on what happens at the other side of the camera and be aware of your surroundings and so that is distracting from what is happening to the patients’ (NO4).

For this nurse, telepresence, (defined by Steuer et al. (1995) as, ‘the experience of presence in an environment by means of a communication medium’ (p. 36), cannot equal physical human interaction (Dolezal et al., 2024). One nurse explained that ‘the reason why I say the patient wants a physical person is that sometimes we just show compassion’. She demonstrated this by extending and arching her arm to perform how she would place it around the shoulders of the patient explaining, ‘I don’t think the robot can do this kind of emotional support for the patient’ (NO5). Another agreed, when operating *Välkky* you cannot ‘touch a patient delicately enough’ (NO6). As Paterson’s (2023) review of robotics and hapticity concludes, ‘hand shaking or hugs’ are deceptively difficult to engineer in human-robot interaction, and ‘touching is unlikely to be simulated in the near term’ (p. 13).

### Meaningful and meaningless robotic care

Two contrasting views of care began to emerge in our data, although both are consistent with long standing images of nursing, rooted in liberal humanism, where a distinction is drawn between highly valued meaning*ful* human-to-human care work, with arguably ‘meaning*less’* mechanical care tasks. Meaning*ful* care practices are also presumed to be more complex requiring a mix of cognitive and emotional expertise, whereas meaning*less* care practices are seen to be unskilled, simple, basic and so readily transferrable. Humanist imaginaries of care as ‘body work’, replete in our data, reproduce this meaning*ful* and meaning*less* care binary. For example, a nurse manager described tasks such as, lifting patients and taking ‘vitals’, as ‘chores’ whereas feeding patients required sensitivity:

‘Our patients have so many difficulties I don’t think robots could do that, but blood pressure, temperature the vitals all of that yes. But not feeding our patients who find it too difficult to swallow’ (HND)

During the focus groups nurses talked enthusiastically about their hope that the robot would ‘take over the more boring aspects of care, . . . the physically loading tasks’ (No3: FG1). Another nurse added, ‘I’ve already heard it can take blood pressure by itself so that and saturation measures would be nice. But I don’t know if it can really help us with the patients which we treat as human beings’ (No4: FG1). However, as we have seen, previous studies of nursing show that articulations demarcating routine and non-routine chores invariably gloss over the subtleties of care work (Latimer, 1995; Traynor, 1996). Indeed, when the nurses began to talk in more detail about their hands-on experiences with *Välkky*, we found their discussions became increasingly nuanced. For example, when asked if *Välkky* could change a catheter the response was:

That’s quite a step forward. If a robot was to change a catheter on me, I would find that very scary because the robot doesn’t have feelings or a knowledge of pain or anxiety (No2). But then again, some people might prefer if it’s a robot doing that . . . its intimate. I don’t know (No1) (FG2).

This exchange about changing a catheter reveals the ‘messiness’ of care (Law, 2004). The nurses see it as a practice that involves cognitive, embodied and emotional skills requiring human-to-human co-presence. It was not a meaningless task, although on reflection they also talked about the potentially intrusiveness of human co-presence. The discussion about inserting a catheter reminds us that the dynamics of care carry multiple meanings. As nurses point out some patients may prefer intimate tasks to be done robotically but others may not. Care work, therefore, initially envisaged as a human versus technology binary, becomes a hybridisation of people, materials, devices and interventions (de la Bellacasa, 2011; Shildrick, 2023).

Nevertheless, a hierarchy of care work is evident from our data with routine and repetitive tasks invariably talked about as less rewarding, more burdensome and requiring little skill. Suchman (1995), writing on technologies in care work, has long argued that it is imperative to move beyond these ‘normative’ notions and instead push for a ‘reflexive engagement’ with care practices (p. 58). The bifurcation of nursing work into complex authentic (skilled human) versus simple assistive (unskilled mechanical) activities risks reinforcing the ‘othering’ of care workers (de la Bellacasa, 2011). Care work thus conceived drains care tasks of their symbolic and cultural meanings, which Brownlie and Spandler (2018) refer to as ‘the relevance of slight’ where small actions such as, handing a cup tea, taking blood pressure, or making a bed are weighty with cultural and emotional significance and are therefore meaning*ful* in how care is understood and valued.

Normative notions of care can, therefore, contribute to the robot figuratively becoming an assistive ‘technician’ to ‘real’ empathic nurses; a stratification of labour that risks foreclosing possibilities of speculating alternative visions of hybridised care. Rather than trying to introduce the robotic nurse as an appendage to assist humans, we propose a *robocentric approach* as an alternative to the extant anthropocentric one. While the latter prioritises human attributes and capacities over other species, *robocentrism* displaces the humanist universe that we so frequently superimpose upon robots. Consequently, we see *Välkky’s* role not as a human assistant but one which harbours potential for the hybridising of care. *Välkky* is not as an assistant to humans, but rather a symbol of potential for the hybridising of care. Considering what is non-human about robots may, for example, be a more appropriate starting point, when appreciating the relationship between their human-like and non-human capacities and limitations. This helps to emphasise their distributed agency and conditionality. *Välkky* as a voyager and actant can disrupt existing, and create novel, ways of caring. We discuss this further, but before we do, let us return to the chronology implicit in our findings, in which nurses’ initial enthusiasm waned when as operators they found *Välkky*’s functionality to be (frustratingly) limited.

## Välkky a Voyager in the Wild

### Trials and tribulations

Enthusiasm articulated in the initial focus group discussions subsided once the training of nurses as teleoperators developed:

Now that I have tried it on the ward, I guess there is nothing especially we can do with it. And it is kind of disappointing. It’s quite awkward it is quite limited what I can do with it, I can’t be helpful or anything like that (NO2).

Another nurse agreed:

Bringing robots to the ward, the hospital setting, is exciting. That I am excited about. But like the practical applications, I am having difficulty at this time trying to see them (NO3).

These comments reflect the realities of operating *Välkky* in ‘the wild’ (Jung and Hinds, 2018: 22), which are particularly resonant because they did not match with the nurses’ expectations, created at the launch event, where ‘everything had worked better’, and ‘the e-skin factor was a very big thing but in real life we were not able to use that function’ (NO4).

Poor internet connectivity limited *Välkky*’s functionality. This was both technical and a human-made problem. The requirement for strong network coverage had been anticipated but subsequently stymied by organisational politics. The HND told us when planning the project, ‘I kept saying a hundred times: “Have you made sure that the network is functioning?” and they all said “yeah, yeah, yeah, yeah” and then when the project team came here, they had no dedicated network’ (HND). When the signal dropped, *Välkky* slowed down or stopped, requiring a ‘restart’ by the trainers. The poor network, worse in the afternoons due to the intensity of other activities on the wards, limited the window for scheduling training sessions. Postponed training impacted on work schedules: ‘It has been a big strain on my work because I have to organise my own day and work so that I have to give extra time for this. My own work doesn’t wait’ (NO3). These problems were further compounded by the fact that the immersive training took longer than had been anticipated, one manager told us they understood that the training would only take a few hours rather than weeks, months or longer.

Our analysis, therefore, indicated that the roles and duties anticipated by focus groups participants no longer seemed possible when using *Välkky* on the ward. Of course, imaginaries are not static but ‘situated’, and shift as the relationship between ‘retrospecting prospects’ (memories of what they expected to happen) and their ‘prospecting retrospects’, which give rise to new expectations (Brown and Michael, 2003). A binary reading of these disappointments could be that the project was a failure, as indicated by the nurses’ frustrations when their expectations about the practical uses of robotic care were not met. However, an alternative interpretation could position these struggles to reconfigure imaginaries, and see them as a resource for enabling the curation of an ontological space and to facilitate the nurses, roboticists, social scientists and philosophers to collaboratively rethink, adapt and revise the robot’s potential.

Informed by her empirical work on care robots Treusch (2017) argues for a ‘queering’ of success versus failure, which in turn encourages us to draw upon multiple forms of knowledge – cognitive, experimental, pragmatic, experiential and affective – that is, ‘touchy-feely styles of thought’ (p. 22). Treusch encourages epistemological and ontological consideration not only about the physics of robotic technology, but also the physics of everyday life, by which we mean the relationalities between time, space, matter, people, meanings and emotions. One nurse, for example, described how patients felt comfortable with *Välkky* when a nurse accompanied it, but were suspicious of it when it was accompanied by the roboticist. He explained:

When I went downstairs with the robot and asked permission if I could visit the patients, I was surprised that the patients responded so positively. Even the more disorientated patients reacted positively and remembered it, some patted it on the shoulder and so on. But when the robot moved alongside [named the roboticist] who had no nurses uniform on, a patient asked, ‘Where is the nurse?’ And that’s why I think, the robot working with a nurse might be better. Once the patients know what the robot is and are familiar with it, then perhaps it can work alone (NO4).

How patients and nurses feel about the robot changes over time. During fieldwork, we saw how patients could be initially hesitant in their encounters with *Välkky*, but on a second or third meeting they began to interact with it more comfortably; chatting to it, teasing it, asking it questions, and offering objects. One patient, for example, handed *Välkky* the TV remote control and another handed over a book she was reading and recommended it to *Välkky*.

*Välkky*, although an ‘object’, it is not necessarily a detached, neutral, singular, inert thing. *Välkky* is reminiscent of another technical device, the bush water pump studied by de Laet and Mol (2000) in their classic paper: ‘The Zimbabwe bush pump: Mechanics of a fluid technology’. Their key message is that the pump is a ‘fluid’ object. This notion of ‘fluidity’ is germane to our observations of *Välkky*. Machines, de Laet and Mol (2000) argue are fluid in three ways. First, the Pump is ‘a mechanical object’ but it is also ‘a device installed by the community’ (p. 252). Second, whether a machine’s ‘activities are successful or not is not a binary matter’, it may for instance work in some situations and not in others, disrupting the binary of success and failure (see also Treusch, 2017 above). In instances where the Bush Pump ‘failed’, opportunities to collaboratively develop adaptions were forged to make the pump fit for purpose. Third, epistemic collaboration between engineers and users means that the technology is regularly ‘tinkered’ with and remade. de Laet and Mol (2000) argue that engineers must be ‘serviceable (or even submissive) to’ (p. 227) users of the Bush Pump if it is to work effectively. We argue that this notion of fluidity is apposite to *Välkky*, which was not, as one nurse put it ‘a technology like so many that are foisted on us’. Rather the robot is a fluid non-human ‘actor’ co-created during a joint voyage of sociotechnical exploration.

We observed how during the trial the roboticists took a ‘serviceable’ (de Laet and Mol, 2000: 227) approach with the nurses. They listened to, and promptly acted on, any difficulties reported by them. Most nurses experienced nausea when using the VR headset. Adjustments were made to the cameras and headsets. These resolved the difficulties. One nurse described auditory problems:

Every time I wear the headset, I can hear the engines running in the robot’s head. I am constantly having to evaluate, ‘Where did that . . . come from?’ To sense where the noise comes from [. . .]. If someone dropped a piece of equipment on the ward, I wouldn’t know which direction it was. And then I just need to use my eyes to locate the sound, then it creates more sound because I can hear the head moving (NO2).

*Välkky* has a ‘digital twin’ in the UK laboratory allowing changes to be implemented overnight with *Välkky* acquiring new ‘body techniques’ (Mauss, 1973 [1934]) by the next morning. All the nurses told us how impressed they were with how all their feedback was acted on so promptly.

## Välkky becomes a sociable robot

### Lively exchanges and somatic responses

One of the nurses noted, ‘the patients are mostly positive towards the robot, and they think it is interesting or funny to see the robot rolling through the door’ (NO3). This reflects Kerruish’s (2021) observation that people can have emotive responses to to robots ‘while simultaneously understanding that they cannot feel or think in the way a human does’ and that some robots have a ‘mechanical liveliness that elicits a response to them as others’.

On the wards, when *Välkky* raises its arms patients commented, ‘it’s angry’, when it shimmies sideways, staff and patients laugh at its ‘dance’ movements, and when *Välkky* waves, patients and staff wave back and chatter informally with it, as recorded in our fieldnotes on ward.

A female patient is in a wheelchair, it is her second time seeing the robot. She stands up and exclaims: ‘Good heavens! I need to see this one standing’. She starts a spontaneous conversation with *Välkky*. She asks the robot: ‘What tasks can you do?’ ‘Can it give medication, and if not, can it learn?’. She asks if it is always this slow in its motion, although she also comments on its agility. Spontaneously the patient suggests tasks that the robot could handle in the future, she mentions lifting patients and burdening tasks. She says, ‘the name *Välkky* (*Bright/clever/smart in Finnish*) fits it perfectly’. And ‘it is fun to greet a robot’. She says, ‘The robot could be the ward’s little beam of light’. (Translators (SM) Fieldnotes).

We might usefully pull on the phenomenological insights of Parviainen et al. (2019) who see a robot as a ‘double body’ which ‘entails both objective and subjective aspects’ generating its ‘aliveness’ (p. 10). Embodied gestures and actions, ‘communicate expressivity irrespective of intentionality’ such that the ‘motions of social robots are not only functional, but they are also emotionally loaded’ (p. 214). This further complicates humanist notions of agency, which rest on the idea of a singular autonomous self, that we touched on above. Shildrick (2023) argues that the agency of robots is relational; they ‘evoke very real emotional and somatic responses and effects, and that alone queers expectations of human capacity’ (p. 129). *Välkky* became a ‘sociable robot’ (Breazeal, 2003). Binaries of human and non-human crumble, as the robot is embedded and entangled in the social, cultural and technical dynamics of the ward (Sumartojo and Lugli, 2022). *Välkky’s* sociability and liveliness emerged.

### Caring for the robot

*Välkky* may be a machine, but a robot Lipp (2023) argues can also become a ‘fragile object of care’ (p. 662). Between morning and afternoon training sessions, a roboticist tells us that ‘*Välkky’s* neck gets tired’ and so it has to ‘rest for a few hours’. When accompanying *Välkky* from the lab to the busy neurology ward via the lift, we hold doors open, request the lift and move rugs to help the teleoperator manoeuvre *Välkky* safely. These are all examples of what Lipp calls ‘mundane courtesies’ (Lipp, 2024: 671). Before entering the ward, *Välkky* has to be ‘gowned up’ with PPE clothing (Image 1 *Välkky gowned*). A designer on the team, used sewing machines available in the city library to make bespoke mitts for *Välkky’s* hands. A patient on the ward by teasing *Välkky*, saying, ‘it was nice to make your acquaintance, the white gown looks good on you, wear it more often!’ (Finnish researcher’s fieldnotes), enhances its agency (Alač, 2016). The ill-fitting gown established *Välkky’s* identity as a novice nurse, thereby substantiating the significance of appearance to social cues through ‘materialities of care’ (Buse et al., 2018).

We have shown how expectations and imaginaries of robotic care altered as *Välkky* travelled through the ward. The presence of robots in the hospital will invariably impact on work schedules, job descriptions, occupational roles, hierarchies, workloads and ward cultures (Pfadenhauer and Dukat, 2015). We must, therefore, proceed slowly and study the intended and unintended consequences of innovative technologies and be alive to their impact on day-to-day care practices. This is neatly captured by a patient in his 80s, who says:

Any nursing task that could assist the nurses in their work is good. But feeding is one of the tasks I wouldn’t want the robot to do. I am so used to nurses providing care, I would find *Välkky* feeding me to be repulsive. The communication between the nurse and the robot is very important. It’s more natural for a nurse to provide the food tray and assist in feeding. But if the robot assisting in the feeding would be *Välkky* and it would be teleoperated I would like to know how it feels, that would make a difference, and I would like to know how it feels. And that is because I could have a discussion with the operator at the same time. (Patient interview - translated).

The patient initially thought that the prospect of being fed by ‘a robot’ ‘would be repulsive’ and yet as he contemplates on how he might feel if *Välkky* was to feed him, he became less adamant. His reflections moved beyond alarmist qualms that robotics will invariably drain care work of its humanity and gave him cause to ponder. This reflects the way that *Välkky* disrupts the binaries of human/non-human, showing how it has the potential to reinforce humanity and affect present in the delivery of even the most intimate parts of care work by health care robots.

## Conclusion

The aim of this paper was to explore *Välkky’s* voyage through a hospital ward, paying particular attention to the study of participant’s expectations and to envisage opportunities and challenges that emerged when working with a care robot in a health care setting. Unlike many previous studies, this world-first ‘trial’ of a unique teleoperated care robot was conducted in a fully operational hospital ward. At the outset, when asked about their expectations relating to the robot, project participants told us how they hoped it could take on routine, mundane and burdensome tasks to free nurses to do ‘real nursing’, by which they meant face-to-face, meaning*ful*, ‘body work’ with their patients. Their imaginaries reflected those found in the policy literatures and in calls to invest in robotic technologies, where there is a view that mundane care tasks can be delegated to machines. However, as training got underway the nurse operators found it difficult to see how the robot could do anything of any use. Yet, as the project progressed and as they worked collaboratively with the roboticists, the nurses’ commitment to *Välkky* rekindled. Reflecting the work of de Laet and Mol (2000) our research demonstrates that an important part of this process was the way in which the roboticists carefully listened to, responded and acted upon the nurses’ feedback. They were quick to implement technical solutions to any problems the nurse operators identified. Through this collaborative working we demonstrated how staff and patients developed a fondness for *Välkky. Välkky* therefore is a fluid object, developing its unique characteristics through interactions with and relations to its social and physical surroundings. Such hybridity lends support to our argument that simply trying to design a robot to take on existing tasks as humans currently do them may be counterproductive. It highlights too the importance of interdisciplinary research on care robotics and the imperative for social science, humanities and engineering to collaborate from the outset. One of the overriding features of this project was the embedding of the robot and the roboticists into an operational hospital and where multiple forms of knowledge – experimental, technical, pragmatic and experiential – where shared. Non-cognitive thought was accepted to be as credible as cognitive thinking.

Working within a post humanist orientation, which envisions a non-anthropocentric vision of care rooted in hybridity, difference and fluidity (DeFalco, 2020; Shildrick, 2023), we proposed a *robocentric approach* which questions the dominant imaginaries of care robotics as potentially replacing, substituting or dispensing with care workers. *Välkky* is a dynamic technology whose functions remain unformed but will coalesce over time. Robots will come to redefine what we understand care to be, as part of their active agency. It is too early to say what care robots can, will or should do, but our findings support the view that rather than looking to identify tasks that we can delegate to robots, we should instead study robotic care in situ, to observe what it looks and feels like, and more importantly, what it achieves in practice. Their distributed agency suggests it is misleading to judge robots, relative to human capacities but rather to assess their role, in defining – and possibly changing – what is regarded as care.

*Robocentrism* encourages us to think about *Välkky* as an actor, a hybrid, a fluid object (de Laet and Mol, 2000) whose presence can disrupt and create new ways of doing care. Deliberations on robotic futures, which see robots as mechanical, objects devoid of ‘humanity’ and as machines that are best suited to menial tasks, would therefore constrain the potential for more creative approaches that move beyond binaries of meaning*ful* and meaning*less* care. A *robocentric approach* to nursing work comprises an evolving process of co-design, where the starting point is what the robot can do, rather than what healthcare professionals or health care managers want it to do. Our findings and subsequent analysis indicate that the nursing care will be continually redefined once robots are in situ. These processes are not linear, but comprise experimentation, reflection and redesign. We might think of *Välkky* as an ‘attractor’ that provokes interest, acting as a dynamic object that contributes to a reimagining of the futures of care.

This has implications for researching care robotics in health settings, we argue it is imperative to sustain a dialogue between health care workers, social scientists and roboticists to ensure that the nuance of any change is captured and understood (Randell et al., 2021). As our research shows, trialling robots must involve embedding them sensitively into real life hospital care settings which are invariably culturally, socially and physically ‘messy’ environments. As the lead roboticist joked when chatting alongside *Välkky* on the hectic, noisy, busy ward ‘we don’t have these problems in outer space’ implying that the messiness of ‘real life’ gets in the way of the imaginaries of what robots can and should do.
